# Exercise-Induced Adaptations to the Mouse Striatal Adenosine System

**DOI:** 10.1155/2020/5859098

**Published:** 2020-01-28

**Authors:** Ella E. Bauer, Trevor J. Buhr, Carter H. Reed, Peter J. Clark

**Affiliations:** ^1^Department of Food Science and Human Nutrition, Iowa State University, 2302 Osborn Drive, Ames, IA 50011, USA; ^2^Neuroscience Program, Iowa State University, 2437 Pammel Drive, Ames, IA 50011, USA; ^3^Interdepartmental Graduate Program in Nutritional Sciences, Iowa State University, 536 Farm House Lane, Ames IA 50011, USA; ^4^Department of Kinesiology, Iowa State University, 534 Wallace RD, Ames, IA 50011, USA

## Abstract

Adenosine acts as a key regulator of striatum activity, in part, through the antagonistic modulation of dopamine activity. Exercise can increase adenosine activity in the brain, which may impair dopaminergic functions in the striatum. Therefore, long-term repeated bouts of exercise may subsequently generate plasticity in striatal adenosine systems in a manner that promotes dopaminergic activity. This study investigated the effects of long-term voluntary wheel running on adenosine 1 (A_1_R), adenosine 2A (A_2A_R), dopamine 1 (D_1_R), and dopamine 2 (D_2_R) receptor protein expression in adult mouse dorsal and ventral striatum structures using immunohistochemistry. In addition, equilibrative nucleoside transporter 1 (ENT1) protein expression was examined after wheel running, as ENT1 regulates the bidirectional flux of adenosine between intra- and extracellular space. The results suggest that eight weeks of running wheel access spared age-related increases of A_1_R and A_2A_R protein concentrations across the dorsal and ventral striatal structures. Wheel running mildly reduced ENT1 protein levels in ventral striatum subregions. Moreover, wheel running mildly increased D_2_R protein density within striatal subregions in the dorsal medial striatum, nucleus accumbens core, and the nucleus accumbens shell. However, D_1_R protein expression in the striatum was unchanged by wheel running. These data suggest that exercise promotes adaptations to striatal adenosine systems. Exercise-reduced A_1_R and A_2A_R and exercise-increased D_2_R protein levels may contribute to improved dopaminergic signaling in the striatum. These findings may have implications for cognitive and behavioral processes, as well as motor and psychiatric diseases that involve the striatum.

## 1. Introduction

The striatum is a component of the basal ganglia that is involved in motor, learning, and motivational processes. Abnormalities in the striatum play a role in a diverse array of neurological and psychiatric disorders, including Huntington's disease, Parkinson's disease, schizophrenia, substance abuse, disordered eating, and depression [1–9]. Evidence indicates that disturbances to energy metabolism may contribute to the onset and progression of neurological and psychiatric disorders that involve striatal dysfunction [10–14]. This is interesting because the striatum may be involved in systemic energy homeostasis [15], as well as the expression of fatigue-related behaviors [16, 17]. Evidence indicates that the striatum is under metabolic demand during aerobic exercise [18, 19], which may disrupt metabolic homeostasis and impair striatal function. Therefore, long-term repeated bouts of physical activity may promote adaptations in factors involved with energy homeostasis to compensate for periods of metabolic demand. Exercise-induced adaptations to metabolic factors within the striatum may have important implications for cognitive and behavioral function, particularly during future energy challenges and neurological disease.

Adenosine signaling within the brain represents a key link between energy homeostasis and neural activity. Indeed, adenosine is a product of adenosine triphosphate (ATP) catabolism that can act like a neuromodulator under periods of metabolic demand [20]. Adenosine receptors are particularly concentrated in the striatum, making it an attractive candidate for exercise-induced adaptations [21–23]. High-affinity adenosine 1 (A_1_R) and adenosine 2A (A_2A_R) receptors are now recognized as potent regulators of striatal circuit activity, in part, due to their postsynaptic location on medium spiny neurons in the striatum [24]. A single episode of exercise can increase brain adenosine concentrations [25]. Therefore, recurrently elevated adenosine levels resulting from repeated bouts of exercise may produce compensatory changes to the expression of striatal A_1_Rs and A_2A_Rs, as increased activity at these receptors can alter ligand binding affinities and cellular protein levels [26–32]. Consistent with this hypothesis, recent evidence indicates that mRNA for A_1_R and A_2A_R receptors become downregulated in the rodent striatum following six weeks of access to running wheels [33], suggesting that physical activity status may also alter receptor properties in the striatum. Potential exercise-induced adaptations to signaling through A_1_Rs and A_2A_Rs could have significant implications for striatal function.

A_1_Rs and A_2A_Rs are G protein-coupled receptors that can also mediate neural activity through their interaction with several neurotransmitter systems (for review, see [34, 35]). However, changes to A_1_R and A_2A_R expression following exercise could have a particularly potent influence on striatal function through its modulation of dopaminergic activity. Indeed, A_1_Rs and A_2A_Rs form heteromeric complexes with dopamine 1 (D_1_R) with dopamine 2 (D_2_R) receptors, respectively [24, 36–40]. Agonist binding at A_1_Rs and A_2A_Rs results in conformational changes to dopamine receptors and decreases dopamine receptor coupling to G-proteins, thereby reducing the effectiveness of dopaminergic signaling within the striatum [39–43]. Impaired dopaminergic signaling in the striatum may favor activity in the basal ganglia pathways that are involved with features of fatigue, including diminished locomotor activity, impaired motivation, and disturbances to executive function [44–50]. Thus, potential exercise-induced changes to the modulation of striatal dopamine activity by adenosine could impact striatum-involved cognitive and behavioral processes, as well as be relevant for managing symptoms of neurological and psychiatric disease related to motor control and motivation. However, whether or not long-term wheel running alters A_1_R and A_2A_R protein expression in the rodent striatum remains unknown.

This study investigates the impact of long-term voluntary wheel running on mouse A_1_R and A_2A_R protein expression in the striatum subregions: the dorsal medial striatum (DMS), dorsal lateral striatum (DLS), nucleus accumbens core (AcC), nucleus accumbens shell (AcS), and lateral nucleus accumbens shell (LAcS), as well as striatal output region: the globus palidus (GP). These striatal subregions were selected because evidence suggests they provide distinct contributions to processing features of affect, motivation, motor behavior, pain, and cognitive function [51–54]. Moreover, equilibrative nucleoside transporter 1 (ENT1) protein density was investigated throughout the striatum, as ENT1 closely balances adenosine concentrations in synaptic space and contributes to the modulation striatum-involved behaviors [55–57]. Finally, exercise-induced changes to striatal D_1_R and D_2_R protein density were also quantified due to their heteromeric interactions with adenosine receptors influencing striatal function [58]. Thus, the capacity of physical activity status to influence A_1_R, A_2A_R, ENT1, D_1_R, or D_2_R protein density in the striatum may be of relevance for understanding the mechanisms by which exercise improves striatum-involved processes and buffers against striatal dysfunction.

## 2. Materials and Methods

### 2.1. Subject and Husbandry

Upon arrival, 32 adult male C57BL/6J mice (Jackson Labs, Sacramento, CA, USA) weighing approximately 25 g were individually housed in standard laboratory cages (35 cm × 30 cm × 15 cm) (*n* = 16) or cages with locked running wheels (35 cm × 30 cm × 15 cm) (*n* = 16). Although stress induced by single housing could potentially impact our dependent measures, mice were singly housed in order to accurately monitor individual running distance. One week after arrival, mice were allowed to run freely (*n* = 16, runners) or kept in standard, nonwheel running cages (*n* = 16, sedentary). Daily wheel revolutions were recorded digitally using Vital View software (Starr Life Sciences, Oakmont, PA, USA). Mice were euthanized either three weeks (*n* = 8 sedentary, *n* = 8 runner) or 8 weeks (*n* = 8 sedentary, *n* = 8 runner). Rooms were controlled for temperature (21 ± 1°C) and photoperiod (12 : 12 L : D) for the entire study. Envigo Teklad 2014 chow and water were provided *ad libitum*. All procedures were approved by the Iowa State University Institutional Animal Care and Use Committee and adhered NIH guidelines. Special care was taken to minimize animal discomfort during all procedures.

### 2.2. Tissue Preparation

Following [59], mice were deeply anesthetized with 100 mg/kg sodium pentobarbital (i.p.) in pairs (one runner and sedentary mouse). Mice were then transcardially perfused with 0.1 M phosphate buffer (PB) solution followed by 4% paraformaldehyde in PB solution. Brains were extracted and postfixed overnight in 4% paraformaldehyde and then transferred to a 30% sucrose solution in PB with saline (PBS) until sectioning. Brains were thinly sliced into 30 *μ*m thick coronal sections using a microtome (Leica SM2010R) with an electronic freezing stage (Physitemp BFS-5MP) set to -22°C. Sections were separated into a 1-in-6 series (i.e., 240-micron increment sections through the rostral-caudal extent of the brain) and stored in tissue cryoprotectant at -20°C until immunohistochemistry.

### 2.3. Immunohistochemistry

Sections chosen for immunohistochemistry for each animal were selected across four locations spanning the rostral to caudal striatum using the Franklin and Paxinos Mouse Brain Stereotaxic Coordinates (4th edition), at approximately 1.54 mm, 0.98 mm, 0.38 mm, and -0.22 mm from the bregma. Immunohistochemistry for each primary antibody was completed on all sections for each mouse simultaneously, using the same solutions for each step. All four sections for each mouse were placed into 1.5 cm diameter net wells. Each wash and incubation period were performed on an orbital rotator for uniformity of solution exposure across all sections. A small number of the striatum-containing sections were incubated with and without (i.e., secondary antibodies only) primary antibodies to support the validity of staining. Furthermore, literature supporting the specificity of primary antibody binding to desired A_1_R, A_2A_R, D_2_, and ENT1 proteins can be found cited in [22, 60–63].

Following [59], the sections were washed three times in 0.1 M PBS for 5 minutes each and treated with 0.6% H_2_O_2_ for 15 min to block endogenous peroxidase activity. They were then washed thrice with 0.1 M PBS for 5 min each time. To block nonspecific binding, the sections were incubated with 5% heat-inactivated PBS-X (in 0.1 M PBS containing 5% goat serum albumin and 0.2% Triton X-100) for 1 h. After blocking was completed, the primary antibody was added for 48 hr incubation times. Primary antibodies were rabbit anti-A_1_R at 1 : 200 dilution (Millipore EMD, AB1587P), mouse anti-A_2A_R at 1 : 600 dilution (Millipore EMD, 05-717), mouse anti-ENT1 at 1 : 1,000 dilution (Santa Cruz Biotech, sc-377283), mouse anti-D_1_R at 1 : 10,000 dilution (Santa Cruz Biotech, sc-33660), or rabbit anti-D_2_R at 1 : 2,000 dilution (Millipore EMD, AB5084P). Both the primary and secondary antibody solutions were diluted in 0.1 M PBS containing 5% normal sera (NGS) and 0.2% Triton-X. Following the primary antibody incubation, the sections were washed with the antibody washing buffer (3% NGS) and 0.2% Triton X-100 in 0.1 M PBS) 4 times for 5 min each time prior to the secondary antibody application. The sections were then incubated using a biotinylated goat anti-rabbit or goat anti-mouse secondary antibody (Vector Laboratories, USA) at a dilution of 1 : 250. The secondary antibody was incubated for 90 minutes at room temperature then the previous washing method was repeated. The sections were then incubated in Vectastain AB solution. Sections were washed again in the same concentration PBS-X solution 4 times with 5 minutes per time. Following washing, the immunohistochemical complex was visualized by exposure to diaminobenzidine and nickel chloride (DABNi, Sigma, USA) for 10 min. The stained sections were rinsed with 0.1 M PBS and then dehydrated in ascending concentrations of alcohol (5 min in 70% ethanol, 5 min in 95% ethanol, 15 min in 100% ethanol, and then 5 min in xylenes), before being coverslipped with mounting medium.

### 2.4. Data Acquisition

A_1_R, A_2A_R, ENT1, D_1_R, or D_2_R protein density was estimated by semiquantitative computer-assisted optical densitometry. Monochrome images of the brain sections were captured digitally using a Leica MC 170 HD mounted camera to a Leica DM4 B digital microscope (Leica Microsystems, Buffalo Grove, IL) at 50x total magnification with constant intensity, exposure, and gain. Images were taken unilaterally alternating right and left hemispheres for each mouse. The corpus callosum (CC) was included as a potential control for regional specificity of physical activity-induced protein changes. However, it should be noted that A_1_R, A_2A_R, and ENT1 mRNA is expressed in oligodendrocytes, so the corpus callosum does not represent a good negative control for these receptors. Relative optical density of brain regions were determined in a blinded fashion using the LAS X software (Leica Microsystems, Buffalo Grove, IL) by placing a square frame of constant size over a region of interest for each brain region (see Table 1 for frame size and stereotaxic coordinates adapted from Franklin and Paxinos Mouse Brain Stereotaxic Coordinates 4th edition). Relative optical density of protein (i.e., light intensity) within the region of interest (see Table 1) was calculated in a computer-generated linear model using LAS X software. Results are reported as relative optical density, which represents the mean signal light intensity within each region of interest.

### 2.5. Statistical Analysis

Trends for A_1_R, A_2A_R, ENT1, D_1_R, and D_2_R relative optical density were consistent across the rostral to caudal sections containing striatal regions. Therefore, relative optical density for A_1_R, A_2A_R, ENT1, D_1_R, and D_2_R protein were reported as an average across the rostral to caudal sections for each striatal subregion.

The relative optical densities for A_1_R and A_2A_R across each striatum subregion were compared using two-way ANOVAs with exercise condition (sedentary vs. runner) and sample time point (three weeks vs. eight weeks) as between-subject factors. *Post hoc* analyses with Fisher's LSD corrections were completed following significant ANOVA interactions between exercise condition and time point.

Tissue availability was limited for three-week sedentary and runner mice, as these sections were used for other analyses (unpublished). Therefore, ENT1, D_1_R, and D_2_R relative optical density was compared only in eight-week sedentary and running mice using unpaired *t* tests. For all analyses, *p* < 0.05 was considered statistically significant. A power analysis was completed on statistically significant results to assure that all comparisons had at least a 0.8 likelihood of detecting an effect that is present.

## 3. Results

### 3.1. Wheel Running

Daily wheel running distance increased steadily for 3 weeks and thereafter maintained a plateau of averaging 7.73 km/d (±0.66 SE) over the final three weeks. The average distance ran for the entire experiment was 7.25 km/d (±0.61 SE). No differences in running distance were observed between mice that ran for three weeks and mice that ran for eight weeks over the first three weeks of wheel access.

### 3.2. A_1_R And A_2A_R Density following Three- and Eight-Weeks of Running

**Figure 1:**
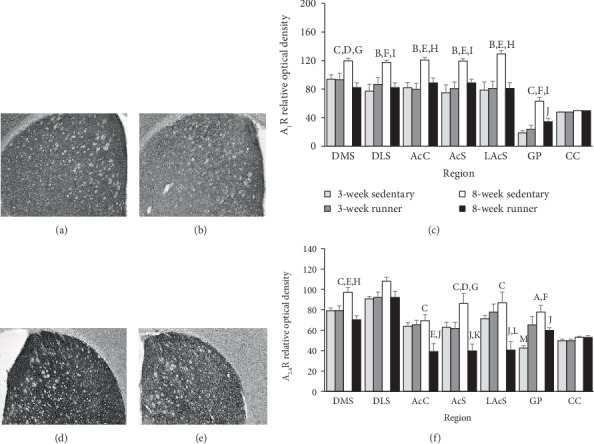
Striatal A_1_R and A_2A_R protein expression following three or eight weeks of wheel running. Representative immunohistochemistry images for A_1_R in the mouse striatum following eight weeks of (a) sedentary conditions and (b) running wheel access. (c) Relative levels of A_1_R protein density represented as averages across sections as detailed in the Methods. Representative immunohistochemistry images for A_2A_R in the mouse striatum following eight weeks of (d) sedentary conditions and (e) running wheel access. (f) Relative levels of A_2A_R protein density represented as averages across sections as detailed in Methods. Statistical significance denoted as follows: sedentary 8 weeks vs. runner 8 weeks at ^A^*p* < 0.05, ^B^*p* < 0.01, and ^C^*p* < 0.001. Sedentary 8 weeks vs. runner 3 weeks at ^D^*p* < 0.05, ^E^*p* < 0.01, and ^F^*p* < 0.001. Sedentary 8 weeks vs. sedentary 3 weeks at ^G^*p* < 0.05, ^H^*p* < 0.01, and ^I^*p* < 0.001. Runner 8 weeks vs. sedentary 3 weeks at ^J^*p* < 0.05. Runner 8 weeks vs. runner 3 weeks at ^K^*p* < 0.05 and ^L^*p* < 0.01. Sedentary 3 weeks vs. runner 3 weeks at ^M^*p* < 0.05.

Representative images for immunohistochemistry staining of A_1_R can be found in Figures 1(a) and 1(b), and for A_2A_R in Figures 1(d) and 1(e) for running and sedentary mice.

Eight weeks of wheel running spared age-related increases of both A_1_R and A_2A_R across the DMS, AcC, AcS, LAcS, and GP. Age-related increases of A_1_R were spared in the DLS following eight-weeks of wheel running; however, this trend marginally failed to reach statistical significance for A_2A_R. There was no statistically significant interaction or effect of exercise condition for A_1_R and A_2A_R receptor density in the CC. However, age mildly increased the expression of both adenosine receptor subtypes in the CC. Test statistics for ANOVAs can be observed in Table 2. Approximate *post hoc* values can be found in Figures 1(c) and 1(f).

### 3.3. ENT1 and Dopamine Receptor Density following Eight Weeks of Running

**Figure 2:**
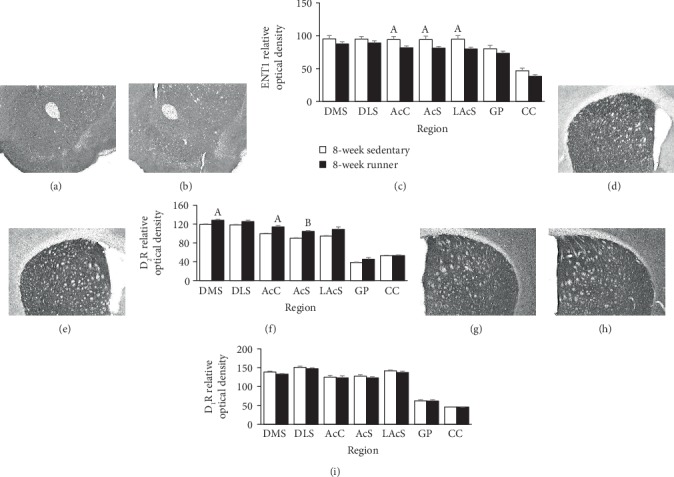
Striatal ENT1, D_2_R, and D_1_R protein expression following eight weeks of wheel running. Representative immunohistochemistry images for ENT1 in the mouse striatum following eight weeks of (a) sedentary conditions and (b) running wheel access. (c) Relative levels of ENT1 protein density represented as averages across sections as detailed in Methods. Representative immunohistochemistry images for D_2_R in the mouse striatum following eight weeks of (d) sedentary conditions and (e) running wheel access. (f) Relative levels of D_2_R protein density represented as averages across sections detailed in Methods. Representative immunohistochemistry images for D_1_R in the mouse striatum following eight weeks of (g) sedentary conditions and (h) running wheel access. (i) Relative levels of D_1_R protein density represented as averages across sections as detailed in Methods. Sedentary 8 weeks vs. runner 8 weeks at ^A^*p* < 0.05 and ^B^*p* < 0.01.

Since differences of protein density for A_1_R and A_2A_R were observed following eight weeks of running, analyses of ENT1, D_1_R, and D_2_R protein density at this time point could provide useful information in the context of observed changes to adenosine receptor protein density. Therefore, analyses were completed following eight weeks of sedentary or running conditions for ENT1, D_1_R, and D_2_R protein density. Representative images for immunohistochemistry staining of ENT1, D_2_R, and D_1_R for sedentary and running mice are, respectively, located in Figures 2(a), 2(b), 2(d), 2(e), 2(g), and 2(h).

Eight weeks of running decreased ENT1 density across the ventral striatum subregions including the AcC [*T*(14) = 2.16, *p* < 0.05], AcS [*T*(14) = 2.17, *p* < 0.05], and LAcS [*T*(14) = 2.57, *p* = 0.02]. However, running produced no statistically significant differences or trends for ENT1 protein density in the DMS, DLS, GP, or CC (Figure 2(c)).

Eight weeks of running increased D_2_R protein density in the DMS [*T*(14) = 2.15, *p* < 0.05], AcC [*T*(14) = 2.67, *p* = 0.01], and the AcS [*T*(14) = 3.45, *p* = 0.004]. A statistically nonsignificant trend towards increased D_2_R protein density was observed in the DLS [*T*(14) = 1.94, *p* = 0.07] and the LAcS [*T*(14) = 1.94, *p* = 0.07]. Running did not impact D_2_R protein density in the GP or CC (Figure 2(f)).

A statistically nonsignificant trend towards decreased D_1_R protein density was observed following eight weeks of running in the DMS [*T*(14) = 1.97, *p* = 0.07]. However, running wheel access did not impact D_1_R protein density in the DLS, AcC, AcS, LAcS, GP, or CC (Figure 2(i)).

## 4. Discussion

The results of this study suggest that eight weeks of wheel running spared age-related increases of A_1_R and A_2A_R protein density across striatal subregions DMS, AcC, AcS, and the LAcS, as well as the striatal output region the GP (see Figure 1). Age-related increases of A_1_R protein density were also spared in the DLS; however, only a similar trend was observed for A_2A_R in this subregion. These findings are consistent with studies suggesting that treadmill running spared age-related increases of A_2A_Rs in the young-adult rat hippocampus [64], and six weeks of wheel running reduced A_1_R and A_2A_R mRNA levels in the adult rat striatum [33]. Eight weeks of wheel running also decreased nucleoside transporter ENT1 protein density notably in the ventral striatum structures AcC, AcS, and LAcS (see Figure 2(c)). At the eight-week running time point, striatal D_1_R and D_2_R displayed distinct patterns of expression from their heteromeric adenosine receptor counterparts. Indeed, D_2_R protein density was subtly increased by wheel running in the DMS, AcC, and AcS compared to sedentary mice (see Figure 2(f)), despite overall trends towards greater density in other striatal subregions that failed to reach statistical significance. This finding is consistent with several reports of mildly increased D_2_R mRNA levels, protein density, and ligand binding affinity in the striatum following periods of exercise [65–71]. Finally, physical activity status had no impact on the density of D_1_R protein (see Figure 2(i)), which has also been reported in previous literature [70, 71]. The immunohistochemistry assessment of protein density provides some evidence that striatal A_1_R and A_2A_R concentrations are affected by physical activity status, which could reduce antagonistic heteromeric interactions with dopamine receptors in the striatum. Together, these data provide a potential mechanism in support of exercise-facilitated dopaminergic function in the striatum [67, 72–74].

An exercise-facilitated dopaminergic function that is consistent with observed changes to A_1_R, A_2A_R, and D_2_R receptor protein densities could alter the activity of striatal circuits in manners that promote locomotor activity, improve affect, and buffer against fatigue-related behavior, especially during challenges to energy homeostasis [72]. The principal neuron of the striatum, the medium spiny neuron, comprises two distinct neural circuits within the striatum, the direct and indirect pathways. Data from studies using gene modification and pharmacological approaches in conjunction with rodent behavior suggest that activation of the direct pathway contributes to reward and ambulation, whereas the indirect pathway activity contributes to aversion and stagnation [75–79]. Differences in the degree of direct and indirect pathway activation, thus, may underlie the varying expression of hedonic and ambulatory states during exposure to pleasurable or noxious stimuli [77]. Interestingly, through antagonistic A_1_R-D_1_R and A_2A_R-D_2_R heteromeric receptor complexes [36, 37, 40, 41, 80], adenosine can reduce postsynaptic D_1_R-mediated activation of direct pathway and D_2_-mediated inactivation of indirect pathway neurons in the striatum [40]. Therefore, the net effect of increased dopamine and adenosine levels acting on exercise-induced reductions of A_1_Rs and A_2A_Rs, as well as increases of D_2_Rs in the striatum, could be to potentiate direct pathway and reduce indirect pathway neuron activity, thereby favoring positive hedonic states and locomotor activity [40], especially during episodes of heightened metabolic demand.

In light of this hypothesis, evidence for greater direct pathway and attenuated indirect pathway activity has been reported in physically active, compared to sedentary, rats during exposure to a series of uncontrollable tail shocks (i.e., acute stress) [33]. Evidence indicates that exaggerated neural pathway activity resulting from exposure to this acute stressor produces widespread energy imbalances in the brain, thereby promoting a state of fatigue, which is a core symptom of depression [13, 81–85]. In fact, antagonism of adenosine receptors in the brain following exposure to acute stress can prevent impaired performance on the shuttle box escape task [81–83], which involves the striatum and is thought to model depression-like motivation deficits [82, 86–88]. Interestingly, six weeks of wheel running access also spares the development of depression-like shuttle box escape deficits in rats exposed to acute stress [89–91], suggesting that exercise may create plasticity in the striatal adenosine system that may contribute to the prevention of stress-induced motivation deficits. The observed exercise-induced changes to A_1_R, A_2A_R, and D_2_R protein densities are consistent with a reduced adenosine-mediated inhibition of striatal dopamine activity, which could favor the stimulation of direct pathway neurons. Greater direct pathway and less indirect pathway activity could contribute to positive emotional states, thereby buffering against motivation deficits resulting from periods of heightened energy expenditure, like exposure to acute stress. However, the modulation of neural activity in the striatum is complex and involves coordinated activity across several neurotransmitter systems and brain pathways. Moreover, immunohistochemical detection of changes to A_1_R, A_2A_R, and D_2_R protein density alone are not sufficient to provide convincing support for these observations. Therefore, future studies are required to determine if a causal relationship exists between these observations.

ENT1 is an integral protein responsible for the transportation of nucleosides, like adenosine, across cellular membranes [92]. ENT1 can mediate adenosine activity by bidirectionally regulating adenosine diffusion across several tissues, including within the central nervous system [56]. Given that ENT1 regulates adenosine flux both into and out of cells, it is not entirely clear how a potential small reduction of transporter protein (see Figure 2(c)) might influence overall adenosine activity in the striatum. On one hand, less available ENT1 could lower the amount of accumulating intracellular adenosine (e.g., during heightened neuron activity) that is transported into synaptic space, thereby limiting the adenosine-related modulation of neuron activity. On the other hand, a potential downregulation of ENT1 protein could also lead to a less efficient removal of adenosine from extracellular space, thereby potentiating adenosine activity at neural circuits. Any potential changes to intra- and extracellular adenosine concentrations by reduced ENT1 protein availability, thus, may be situational and contingent on the sources and amounts of accumulating adenosine. Moreover, extracellular adenosine concentrations are regulated through multiple mechanisms of transport and metabolism enzymes (e.g., adenosine kinase and adenosine deaminase), and therefore cannot be completely accounted for by the potential reduction of a single transporter protein [20, 56, 92, 93]. A more complete analysis of enzymes that metabolize adenosine and nucleoside uptake proteins must be considered to fully understand the influence of physical activity on the regulation of adenosine concentrations in synaptic space.

In conclusion, the current data provide novel evidence that exercise promotes adaptations in the striatal adenosine system. Reductions to A_1_R and A_2A_R protein expression could contribute to reduced efficacy of adenosine-mediated inhibition of dopamine activity in the striatum, which should be followed up in more detail by future studies. Indeed, changes in A_1_Rs and A_2A_Rs that are dependent on physical activity status could affect therapeutic approaches for psychiatric and neurological diseases that involve abnormal dopamine signaling in the striatum, including addiction, depression, Parkinson's disease, and Huntington's disease, among many others [94–104]. Therefore, the current findings could be of importance for understanding the mechanisms contributing to exercise-improved cognitive function, as well as the prevention and treatment of mental health and neurobiological disorders that involve the striatum.

## Figures and Tables

**Table 1:** Stereotaxic coordinates and frame size used for densitometry.

Section	Region	Frame (microns)	Bregma (mm)	M/L (mm)	D/V (mm)
1	DMS	400 × 400	1.54	±0.8	3.0
2	DMS	400 × 400	0.98	±1.0	3.2
3	DMS	400 × 400	0.38	±1.1	3.3
4	DMS	400 × 400	-0.22	±1.7	3.6
1	DLS	400 × 400	1.54	±2.0	2.8
2	DLS	400 × 400	0.98	±2.2	2.8
3	DLS	400 × 400	0.38	±2.6	2.8
4	DLS	400 × 400	-0.22	±3.0	2.5
1	AcC	250 × 250	1.54	±1.0	1.1
2	AcC	250 × 250	0.98	±1.2	1.0
1	AcS	250 × 250	1.54	±0.8	1.0
2	AcS	250 × 250	0.98	±0.8	0.8
1	LAcS	250 × 250	1.54	±1.8	1.0
2	LAcS	250 × 250	0.98	±2.0	0.8
4	GP	400 × 400	-0.22	±1.8	2.0
1	CC	75 × 75	1.54	±1.0	3.6
2	CC	75 × 75	0.98	±1.0	3.8
3	CC	75 × 75	0.38	±1.0	3.8
4	CC	75 × 75	-0.22	±1.0	3.8

**Table 2:** List of ANOVA results for adenosine 1 and adenosine 2A receptor protein density in striatal subregions. ^∗^*p* < 0.05 interaction between time point and condition.

Region	Receptor	Time point	Exercise condition	Interaction
DMS	A_1_R^∗^	*F*(1, 28) = 1.22, *p* = 0.280	*F*(1, 28) = 7.90, *p* = 0.009	*F*(1, 28) = 7.30, *p* = 0.012
	A_2A_R^∗^	*F*(1, 28) = 1.26, *p* = 0.270	*F*(1, 28) = 11.00, *p* = 0.002	*F*(1, 28) = 11.26, *p* = 0.002
DLS	A_1_R^∗^	*F*(1, 28) = 5.38, *p* = 0.028	*F*(1, 28) = 2.79, *p* = 0.106	*F*(1, 28) = 8.34, *p* = 0.007
	A_2A_R	*F*(1, 28) = 3.70, *p* = 0.065	*F*(1, 28) = 2.48, *p* = 0.127	*F*(1, 28) = 3.61, *p* = 0.068
AcC	A_1_R^∗^	*F*(1, 28) = 12.13, *p* = 0.002	*F*(1, 28) = 6.14, *p* = 0.019	*F*(1, 28) = 4.62, *p* = 0.040
	A_2A_R^∗^	*F*(1, 28) = 3.22, *p* = 0.084	*F*(1, 28) = 5.98, *p* = 0.021	*F*(1, 28) = 7.38, *p* = 0.011
AcS	A_1_R^∗^	*F*(1, 28) = 11.12, *p* = 0.002	*F*(1, 28) = 2.49, *p* = 0.126	*F*(1, 28) = 5.35, *p* = 0.028
	A_2A_R^∗^	*F*(1, 28) = 0.01, *p* = 0.924	*F*(1, 28) = 10.41, *p* = 0.003	*F*(1, 28) = 9.58, *p* = 0.005
LAcS	A_1_R^∗^	*F*(1, 28) = 7.85, *p* = 0.009	*F*(1, 28) = 6.51, *p* = 0.016	*F*(1, 28) = 7.93, *p* = 0.009
	A_2A_R^∗^	*F*(1, 28) = 1.73, *p* = 0.200	*F*(1, 28) = 5.89, *p* = 0.022	*F*(1, 28) = 10.34, *p* = 0.003
GP	A_1_R^∗^	*F*(1, 28) = 33.28, *p* < 0.001	*F*(1, 28) = 6.17, *p* = 0.019	*F*(1, 28) = 12.31, *p* = 0.002
	A_2A_R^∗^	*F*(1, 28) = 6.51, *p* = 0.016	*F*(1, 28) = 0.17, *p* = 0.687	*F*(1, 28) = 12.12, *p* = 0.002
CC	A_1_R	*F*(1, 28) = 18.70, *p* < 0.001	*F*(1, 28) = 0.05, *p* = 0.819	*F*(1, 28) = 0.03, *p* = 0.870
	A_2A_R	*F*(1, 28) = 4.55, *p* = 0.042	*F*(1, 28) = 0.00, *p* = 0.993	*F*(1, 28) = 0.00, *p* = 0.995

## Data Availability

The data used to support the findings of this study may be released upon request to Peter Clark at Iowa State University, who can be contacted at pjclark@iastate.edu.
